# Efficient Homologous Recombination in Mice Using Long Single Stranded DNA and CRISPR Cas9 Nickase

**DOI:** 10.1534/g3.118.200758

**Published:** 2018-11-21

**Authors:** Xi A. Ge, Craig P. Hunter

**Affiliations:** Department of Molecular and Cellular Biology, Harvard University, Cambridge, MA 02138

**Keywords:** Cas9 nickase, Long single stranded DNA, ssODN

## Abstract

The CRISPR/Cas9 nickase mutant is less prone to off-target double-strand (ds)DNA breaks than wild-type Cas9 because to produce dsDNA cleavage it requires two guide RNAs to target the nickase to nearby opposing strands. Like wild-type Cas9 lesions, these staggered lesions are repaired by either non-homologous end joining or, if a repair template is provided, by homologous recombination (HR). Here, we report very efficient (up to 100%) recovery of heterozygous insertions in *Mus musculus* produced by long (>300 nt), single-stranded DNA donor template-guided repair of paired-nickase lesions.

Genome editing of mice (*Mus musculus*) greatly contributes to its value as a mammalian model for the study of development, cancer, infectious diseases and other evolutionarily conserved biochemical pathways. Although emerging technologies have eased gene disruption, a parallel increase in the efficiency of gene insertion technology, particularly larger insertions, has not been realized (Doudna and Charpentier 2014; Jiang and Marraffini 2015). Sequence insertion relies on repair of dsDNA breaks (DSB) by homologous recombination (HR) DNA repair pathways using provided donor template DNA. The development of programmable site-specific nucleases, initially zinc finger nucleases (ZFN) and transcription activator-like effector nucleases (TALEN) and more recently CRISPR/Cas9 nuclease, which create targeted dsDNA breaks has greatly increased the efficiency of HR mediated DNA insertion in mouse ES cells and led to the development of similar approaches in mouse embryos. These advances have reduced the cost and time of developing mouse genetic models.

The ease of designing guide RNA targets for the CRISPR/Cas9 nuclease has largely supplanted the other programmable nucleases. The guide RNA includes a 20-nucleotide programmable targeting (protospacer) region that in large genomes may provide incomplete specificity, resulting in off-target cleavage and unwanted alterations (Wang *et al.* 2013; Mali *et al.* 2013; Jinek *et al.* 2013). To reduce off-target effects, a Cas9 nickase (Cas9n) D10A mutant was developed (Ran *et al.* 2013a). Cas9n cleaves only one strand of the targeted DNA, producing a nick, a single-stranded DNA break. Thus, a targeted dsDNA break requires two single guide RNAs (sgRNAs) to target the Cas9n to opposing strands in close proximity (Ran *et al.* 2013a; Shen *et al.* 2014; Mali *et al.* 2013). The probability that Cas9n will nick two non-targeted DNA strands in close proximity is negligible, thus greatly increasing the fidelity of genome modification.

Traditional DSB HR repair templates have been dsDNA fragments or plasmids with very long (1-5 kb) homology arms that flank the insertion site (Mali *et al.* 2013; Rong *et al.* 2014). However, these dsDNA donors can be challenging to design and prepare, particularly if the target region includes repetitive sequences. Long dsDNA donor templates also have a stubbornly low (<= 5%) integration efficiency (Table S1) (Mali *et al.* 2013). In contrast, easily obtained commercial ssDNA oligos (ssODN) up to 200 nt long are associated with higher (>20%) HR efficiency, (Chen *et al.* 2011; Richardson *et al.* 2016), but multiple factors may impact HR efficiency. Additionally, because their homology arms are short, it is easy to use PCR strategies to identify and confirm accurate insertion events. However, their short length limits their use to targeted point mutations, short insertions, or precise deletions (Ran *et al.* 2013a; Wang *et al.* 2013; Mali *et al.* 2013; Ran *et al.* 2013b). In addition, this short length complicates the use of Cas9n, which because of the distance between nick sites reduces the effective length of the homology arms. Long (l)ssDNA donor templates produced by *in vitro* transcription and reverse transcription (*iv*TRT) have been used for HR-mediated repair of Cas9 targeted DSB (Miura *et al.* 2015). The use of lssDNA donor templates not only allows larger insertions, the longer length also better accommodates the extended spacing between paired nick sites, potentially enabling the use of Cas9n with fewer accompanying off-target mutations.

Here we report that lssDNA, containing numerous donor template mutations to eliminate re-targeting events, is very efficiently inserted at Cas9n targeted genes. We simultaneously introduced into 1-cell mouse embryos sgRNAs and lssDNA donor templates targeting two unlinked genes for C-terminal immune-tag insertions. Among 32 live born mice, we recovered 32 insertions at the first locus and 12 insertions at the second. Curiously, all 44 insertions were recovered in an apparent heterozygous state. Furthermore, at the first locus, while the intended PAM-disrupting mutation was successfully introduced for one of the sgRNAs, all insertion events failed to include the stretch of synonymous mutations in the protospacer for the other sgRNA. Together these observations indicate that either Cas9n-produced staggered DSB and/or lssDNA repair templates may employ a non-standard HR repair mechanism.

## Materials and Methods

### Database and Software

The CHOPCHOP database was used to identify potential sgRNA binding sites near the *Sidt1* and *Sidt2* stop codons (Montague *et al.* 2014). The top three for each gene were selected as candidates for verification in NIH3T3 cells.

### Oligo, gBlock, Primers and Cas9n mRNA

Commercial *Sidt1* and *Sidt2* single-stranded donor DNA oligos (Table S3), gBlocks for sgRNA and lssDNA synthesis (Table S4) and all primers (Table S5) were ordered from Integrated DNA Technologies (IDT), Inc. Eukaryotic CRISPR/Cas9n (D10A) mRNA was obtained from System Biosciences, Inc. (SBI).

### sgRNA Synthesis, Purification, and Surveyor activity assay

Synthesized sgRNAs sequences (gBlocks, IDT) were inserted into expression plasmids, sequenced verified, and tested for activity in NIH3T3 cells. To construct plasmids, sgRNA sequences with a eukaryotic promoter synthesized as a gBlock (Table S4) from IDT were PCR amplified using high-fidelity Phusion Master Mix (Thermo) and gel-extracted, dA-tailed, ligated to pCR4-TOPO (Invitrogen) and transformed into NEB10β cells. Single colonies were cultured in LB-broth for sequence verification. hCas9 (Addgene) and gRNA plasmids for transfection were purified using the Qiagen EndoFree Plasmid Mini Extraction Kit. Cas9 and the sgRNA vectors were co-transfected with Lipotransfectamine 2000 (Invitrogen) into NIH3T3 cells and cultured for three days before lysis and genomic DNA preparation. PCR products using *Sidt1* and *Sidt2* surveyor primers (Table S5) on positive and negative control (empty gRNA vectors) DNA samples were denatured and then renatured either alone or as a 1:1 mix before treatment with Surveyor nuclease and analysis by gel electrophoresis per manufacturer’s instructions (Transgenomic). All gel electrophoresis used 2% agarose (SeaKem) with 0.5 µg/ml ethidium bromide prepared and run in TBE buffer.

For microinjection preparations, primers with T7 promoter and terminator extensions (Table S5) were used to amplify (Phusion Master Mix, ThermoFisher) the sgRNA sequences from each plasmid. These DNA fragments were then *in vitro* transcribed into sgRNAs (AmpliScribe T7-*Flash* Transcription Kit, Epicentre), treated with DNase I, purified (RNeasy Mini Kit, Qiagen), precipitated by 2.5 M NH_4_Ac (Sigma) and intensively washed with 70% ethanol (at least 6 times), dried and dissolved in molecular grade H_2_O (Corning). Concentration and purity were evaluated by electrophoresis and spectroscopy (NanoDrop).

### Long ssDNAs Synthesis and Purification

Synthesized lssDNA donor sequences (gBlocks, IDT) were inserted into plasmids, sequence verified, and PCR-amplified using primers with a T7 promoter extension but no terminator (Table S4). The DNA amplicons were then *in vitro* transcribed as above into RNA and treated with DNase I. The RNA transcripts were then reverse transcribed into cDNA (ThermoScript RT-PCR System, Invitrogen) using the 5′ primer. To remove RNA from the resulting lssDNA the samples were first treated RNase H and then subjected to alkaline hydrolysis by suspension in 100 mM NaOH, 10 mM EDTA at 70° for 20 min and then neutralized by adding 1/3 volume 0.5M Tris-HCl (pH 6.4). To precipitate the ssDNA, the sample volume was adjusted to 400 μl, and 40 μl 3 M NaAc (pH 5.2) and 1 ml 100% ethanol were added. The mixed sample was then incubated at -20° for 30 min and centrifuged (14,000g) for 15 min. The pellets were washed (6X) with 70% ethanol, air dried and dissolved in molecular-grade water.

### Micro-Injection into Mouse Zygotes and Fostering

Micro-injections of all ssDNAs, sgRNAs and the eukaryotic Cas9n mRNA, as well as the fostering were done according to the previously established methods (Yang *et al.* 2014; Wang *et al.* 2013; Mali *et al.* 2013) at the Genome Modification Facility (GMF) of Harvard University. Briefly, ∼300 E0.5 C57BL/6 zygotes were micro-injected and cultured in KSOM medium at 37° in a 5% CO_2_ incubator for 24 hr (two-cell stage). 150 embryos were transferred into 5 BALB/c surrogate mothers via their oviduct. The pups were born after about 20 days and lactated by the same mothers for additional 21 days until they are weaned and genotyped.

### Mouse Breeding and Genotyping

All breeding was performed at the Biological Resource Infrastructure (BRI) of Harvard University. The F_0_ mouse 2 was systematically mated with many female wild-type mice to obtain allele frequency distribution data. Genotyping is based on PCR of gDNA isolated from tail tissues of F_0_-F_3_ mice with primers specific to each locus.

### Data Availability

The authors affirm that all data necessary for confirming the conclusions of this article are represented fully within the article and its tables and figures. Supplemental material available at Figshare: https://doi.org/10.25387/g3.7315478.

## Results

### Commercial oligo directed repair of Cas9n-produced DSB

The mammalian *Sidt1* and *Sidt2* genes are homologous to *C. elegans* SID-1, a large transmembrane protein that imports extracellular dsRNA into the cytoplasm (Feinberg and Hunter 2003). To investigate the expression and localization of the mouse *Sidt1* and *Sidt2* genes we designed and ordered commercially synthesized 200 nt long ssDNA oligodeoxynucleotides (ssODN) to insert carboxy-terminal Myc_(3X)_ and HA_(3X)_ epitope tags at the C-terminus of both genes (Figure S1). To limit potential off-target mutations we used Cas9n. We used the CHOPCHOP database (Montague *et al.* 2014) to identify candidate gRNAs and the Surveyor assay (Hsu *et al.* 2013; Ran *et al.* 2013a) to select two active sgRNAs (Figure S2) targeting opposing strands for each gene (Table S2). The selected *Sidt1* sgRNAs flank the intended insertion site while the *Sidt2* sgRNAs are both 5′ to the insertion site (Figure S1). The *Sidt1* 200 nt repair template contained third-position PAM mutations at both sgRNA1 and sgRNA2 binding sites, while *Sidt2* sequence constraints limited PAM mutations to the second position for sgRNA4 (Table S3). Only a portion of the sgRNA3 binding site was included in the 200 nt oligo (Figure S1).

These four *in vitro* synthesized sgRNAs and the two ssODN were microinjected along with eukaryotic Cas9n mRNA into C57BL/6 zygotes, which were then transplanted into 5 BALB/c surrogate mothers. 13 live pups were recovered and genotyped for *Sidt1* and *Sidt2* insertions. PCR genotype showed either a WT sized band or slightly smaller for most pups, but a single female (oligo mouse [om]11) was identified with a likely mosaic insertion at the *Sidt1* locus (Figure S3); however, this female died of dystocia before generating any live offspring. Genotype analysis of the dead pups confirmed the presence of the expected insertion, a truncation allele, and a wild-type locus (Figure S3). Three mice (om1, om2, om8) were recovered with insertions at the *Sidt2* locus (Figure S3). However, PCR and sequence analysis indicate that multiple repeat insertions had occurred within the chromosome of each mouse, producing complex insertions at the locus, (data not shown). This likely reflects the lack of an effective PAM site mutation within the sgRNA binding sites of that ssODN. To enable the inclusion of sgRNA binding site mutations in the repair template we modified our approach to use lab-synthesized long (l)ssDNA donor templates.

### Design, synthesis, and purification of long ssDNA donor templates

**Figure 1:**
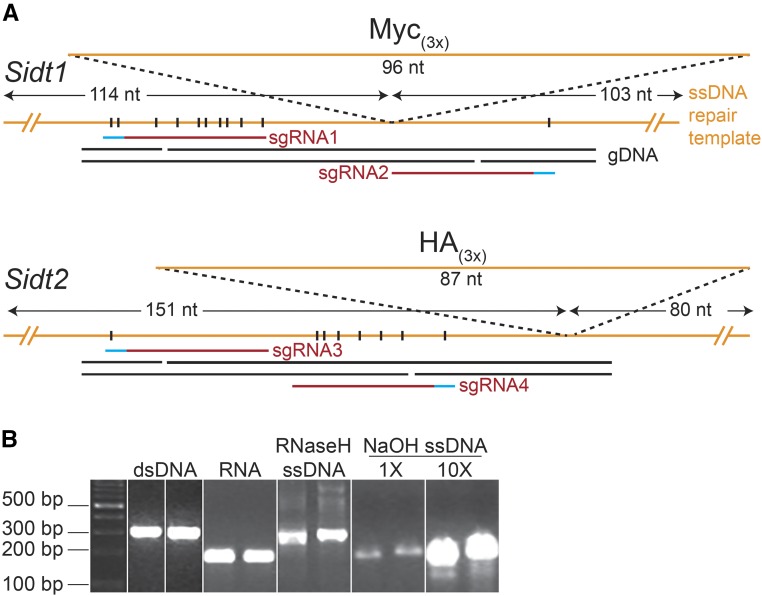
Design, synthesis and purification of long ssDNA donors and sgRNAs. A) sgRNA1/2 (red) flank the *Sidt1* insertion site (IS), while sgRNA3/4 are both 5′ of the IS. The resulting Cas9n produced single-stranded nicks on opposing genomic DNA (black) are indicated. The *Sidt1*-Myc_(3X)_ long ssDNA donor (orange) includes 103 nt and 114 nt flanking homology arms (measured from IS), and multiple silent mutations (vertical black lines) in sgRNA1 target (red) and PAM (blue) site and a single mutation in sgRNA2 PAM. The *Sidt2*-HA_(3X)_ long ssDNA donor includes 80 nt and 151 nt flanking homology arms, a single PAM mutation in sgRNA3, and multiple silent mutations in the sgRNA4 and PAM site. The perfect-matching 5′/3′ homology arms are 77-/80-nt for *Sidt1* and 80-nt for *Sidt2*. B) PCR-amplified *Sidt1* and *Sidt2* dsDNA (336/341-bp, including a T7 promoter) and the corresponding RNA products after *in vitro* transcription. The RNaseH-treated *in vitro* transcription reverse transcription products (expected molecular sizes of 313/318-nt) unexpectedly migrated as a double-stranded product, similar to the dsDNA template for RNA synthesis. The migration of alkaline hydrolysis (100 mM NaOH) treated RT products (1X and 10X) was, as expected for a ssDNA molecule, more similar to the ssRNA templates. Note: images cropped and resampled (see Figure S5 for originals).

We designed lssDNA donor templates with longer homology arms that included multiple mutations in the sgRNA binding sites (Figure 1A, Table S5). To synthesize these lssDNAs, we used an *in vitro* transcription and reverse transcription method (*iv*TRT) (Miura *et al.* 2015). Specifically, we used reverse transcriptase to produce a long single-strand cDNA from RNA transcribed from a PCR amplicon generated from sequence-verified, custom synthesized dsDNA (IDT gBlock) (Figure 1B). The addition of an alkaline hydrolysis step and quantitative precipitation (methods) resulted in a near 100% conversion and recovery of ssRNA into ssDNA, 15-fold higher recovery than the previous report (Miura *et al.* 2015) and analysis of the product indicates high purity and lack of residual RNA fragments (Figure 1B). Both lssDNA products were verified by Sanger sequencing (data not shown).

### Genotyping and sequencing revealed highly efficient but incomplete insertions

**Figure 2:**
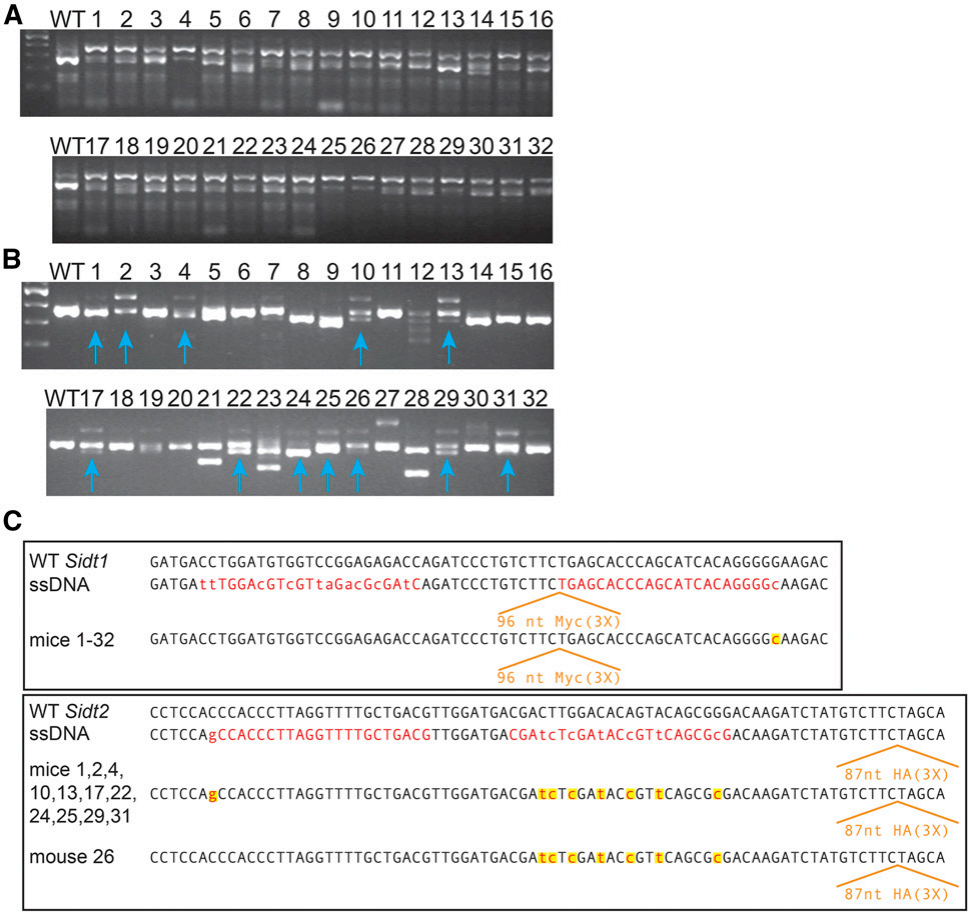
PCR genotype analysis of F_0_ mice. PCR analysis (tail biopsies) shows that 32/32 and 12/32 F_0_ mice contain a PCR product of the expected size for an insert at the *Sidt1* (A) and *Sidt2* (B) loci respectively. Vertical arrows indicate candidate *Sidt2* insertions. All these candidate knock-in bands were confirmed by sequencing gel purified-presumptive insert bands (data not shown). (C) *SidT1*-Myc_(3x)_ and *Sidt2*-HA_(3X)_ sequence results from F_0_ Mice. The tail biopsy PCR product of the expected size for an insert (Figure 2) was gel purified and sequenced. The wild-type (WT) sequence and the sequence of the ssDNA donor is shown for reference. All 32 recovered mice showed identical sequence at the *Sidt1* locus. Eleven of the 12 mice with a proper insertion at the *Sidt2* locus incorporated all ssDNA donor mutations, while one mouse was WT for the most 5′ mutation. Note: images cropped and resampled (see Figure S5 for originals).

We injected the two new lssDNAs, with the previously designed sgRNAs and Cas9n mRNA into C57BL/6 zygotes and recovered 32 F_0_ pups. We note the significantly larger litter sizes (avg. 6.4) compared to the commercial oligo-injected group (avg. 2.6). PCR genotyping revealed that all 32 pups (17 males and 15 females) produced a PCR product of the expected size for a single copy Myc_(3X)_ tag insert at the *Sidt1* locus (Figure 2A), while 12 pups (10 males and 2 females) produced a PCR product of the expected size for a single copy HA_(3X)_ tag insert at the *Sidt2* locus (Figure 2B). This insertion rate per pup is more than 10-fold higher than the commercial oligo injected group. We next sequenced gel extracted DNA bands to verify in-frame Myc_3X_ tag and HA_3X_ tag inserts (Figure 2C). The sequence analysis confirmed that all the engineered mutations in both *Sidt2* sgRNA binding sites were introduced into 11 of 12 mice with inserts (one mouse failed to confer the 5′ most PAM mutation). In contrast, for all 32 mice with proper inserts at the *Sidt1* locus only the 3′UTR PAM2 mutation was present; in no mice were any of the sgRNA1 binding site mutations present (Figure 2C). This indicates that the 5′ 100 nt region of the donor template was not included in the repaired chromosome and that the upstream recombination site (gene conversion track) must be within the 14-nt region between the HA_(3X)_ tag insert site and the target sgRNA1 binding site (protospacer) mutations (Figure 1A). It is possible that the multiple binding site mutations disrupt pairing, thus interfering with HR repair of the lesion. However, analysis of the single recovered mosaic female showed that the 5′ single-third position PAM mutation encoded in the commercial oligo, also failed to incorporate into the *Sidt1* locus (Figure S4A). Thus, it is likely that the exclusion of the 5′ donor sequences from the repaired locus reflects a consequence of the unique structure of the DNA lesion and/or the ssDNA donor template.

### Segregation of induced alleles is consistent with recovery of heterozygous mice

**Figure 3:**
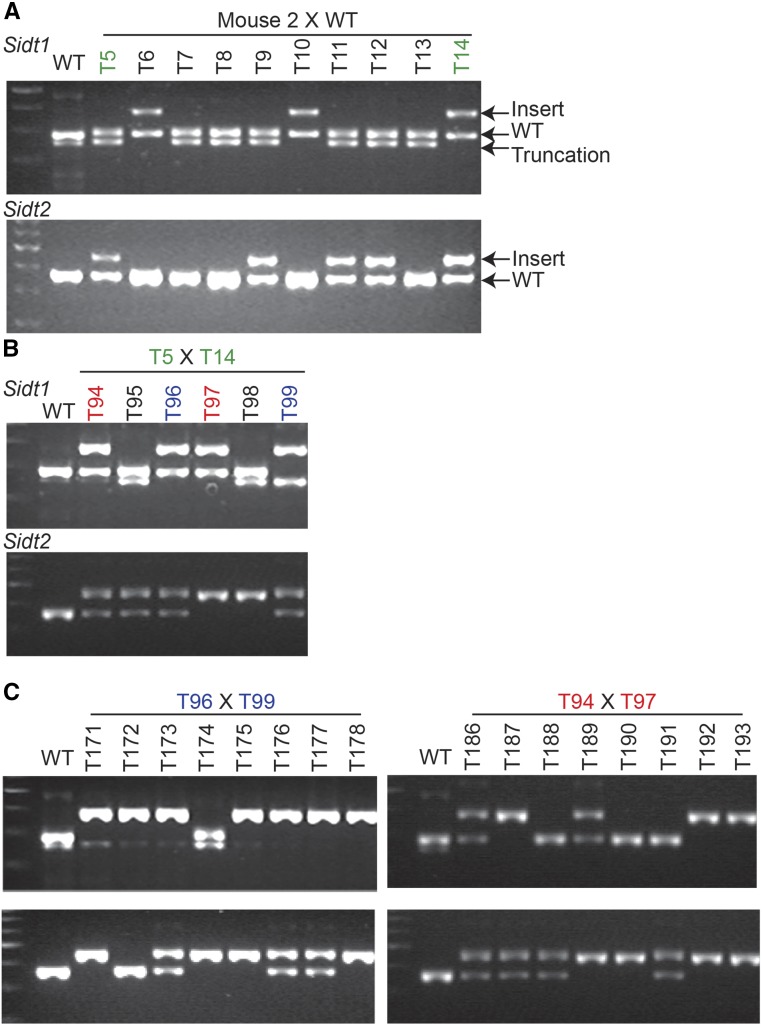
Segregation of insertions and truncations at the *Sidt1* and *Sidt2* loci. The genotyping results for F_1_ (A), F_2_ (B), and F_3_ (C) both *Sidt1* (upper gel) and *Sidt2* (lower gel) loci, with the insert, and wild-type alleles of both genes as well as the truncation allele only for *Sidt1*, as indicated on the right. Note: images cropped and resampled (see Figure S5 for originals).

Select *Sidt1-Myc_3X_* and *Sidt2-HA_3X_* insertions were successfully bred to homozygosity at the F_2_/F_3_ generation, displaying expected Mendelian ratios, consistent with either F_0_ heterozygosity or approximately 1:1 germline mosaicism (Figure 3). Indeed, the F_1_ mice proved to be heterozygous for either the targeted insertion or an indel at the locus (Figure 2), indicating that these F_0_ mice were heterozygotes. Sequence analysis of the *Sidt1* indel associated with mouse 2, recovered together with lssDNA injections, indicates a deletion corresponding to a portion of the region between the two nicks and the insertion of a 10 bp inversion of the deleted region (Figure S4B). This indicates that the long single-stranded 5′ overhang was incompletely resected prior to donor independent repair. Thus, the structure of the DSB (48 nt long 5′ overhangs) may impact both NHEJ and HR repair pathways.

## Discussion

Here we report that Cas9n-produced dsDNA breaks paired with long-ssDNA donor repair templates resulted in highly efficient and readily detected and verified insertions in mice. To accommodate two sgRNA targets and an epitope-tag insertion requires a repair template longer than those currently available by direct chemical synthesis of ssDNA oligos. Rather than using a long dsDNA, which are relatively inefficient repair donors and necessitate extensive flanking homology regions that complicate detection and verification of insertion events, we used *in vitro* reverse transcription of an RNA template to produce a lssDNA repair template. Because the flanking homologous regions were reasonably short, we were able to design primers external to the donor sequence and use simple PCR to identify and verify insertions at the target locus. All recovered mice contained one or more proper insertions.

Accompanying the very high insertion frequency (32/32 and 12/32) was the persistent recovery of only heterozygous or mosaic mice. In many cases the insertion on one chromosome was accompanied by a non-templated lesion on the other. Indeed, both sets of our experiments with Cas9n, the ssODN and lssDNA, produced apparently heterozygous mice, while similar experiments with Cas9 did not (Miura *et al.* 2015; Wang *et al.* 2013; Mali *et al.* 2013; Codner *et al.* 2018; Lanza *et al.* 2018).

Mouse paternal and maternal chromosomes differ in chromatin states (van der Heijden *et al.* 2005), which is known to affect DNA repair (Watts 2016). It is also reasonable to assume that different paternal/maternal heterochromatin states may limit access to Cas9 or Cas9n, leading to preferential double-nicking of either the maternal or paternal chromosome. Thus, a simple explanation for the exclusive recovery of heterozygous insertions is that paternal and maternal chromosomes differentially accessible to either Cas9n or to repair (HR *vs.* NHEJ) of double-nicked DNA. Similar experiments in mice with distinct parental polymorphisms near the target locus could provide insight.

We also note, that at the *Sidt1* locus all 32 insertions failed to incorporate any donor template mutations more than 14 nt 5′ of the insertion site, effectively a 14 nt 5′ homology region. This unexpected outcome may result from either the large number of pairing disrupting mutations in the protospacer region and/or the observation that ssDNA repair templates may use the Fanconi Anemia-dependent but not the Rad51-dependent pathway (Richardson *et al.* 2018). It is also unknown whether this is related to the non-canonical HR pathway observed in the single-nick repair process (Davis and Maizels 2014). Resolution of these issues will require biochemical and genetic analysis.

Using a sequence-verified template, our improved *in vitro* long ssDNA synthesis and RNA removal protocol produces a high yield of pure ssDNA free of endotoxins and contaminating RNA products. This allowed us to inject the mouse zygote with multiple repair templates at a high concentration. Compared to similar injection of chemically synthesized moderately long oligos, we observed a significant increase in pup production. It is possible that chemically synthesized oligos are contaminated with incomplete synthesis products or residual nucleotide-protecting residues that would be absent from the *in vitro* synthesized lssDNA. In addition, our improved method for lssDNA purification and recovery removed residual RNA fragments and produced much higher yields than the previous report (Miura *et al.* 2015). In *C. elegans*, ssDNA donor purity and high concentration is essential for efficient HR (Ward 2015), thus the high concentration of pure lssDNA may also provide an explanation for our observed high efficiency. Several studies using nuclease resistant nucleotides show that end-modified ssODN exhibit enhanced intracellular stability that correlates with efficiency (Renaud *et al.* 2016; Liang *et al.* 2017). Thus, the lssDNA may as well simply be a larger substrate, preserving essential homology. Higher DNA concentration may function by the same principle. Furthermore, this approach eliminates the sequence errors associated with chemically synthesized long oligos that may interfere with HR and introduce unwanted changes into the genome. The longer length also allows a more extensive introduction of silent mutation within protospacer and PAM sites; this flexibility is particularly important when designing inserts at/near coding regions or exon/intron boundaries.

The use of Cas9n compared to Cas9 introduces the design constraint of a second functional sgRNA binding site, which may be at some distance from the first. However, published work shows that Cas9n sites can be 100 bp apart and support efficient NHEJ (Ran *et al.* 2013a). In our case, the 5′ overhangs 22-48 long supported quite efficient recombination of donor sequences. The relatively minor increase in time to prepare long ssDNA from commercial gBlock DNA is more than compensated by the reduced number of mice that need to be produced and screened by simple PCR and sequencing. If the high frequency of recovered heterozygous insertion and indel alleles is a general feature of lssDNA repair of paired-nick sites, then this may be an ideal approach for precise genome editing in mice.
